# Supplementary material for: Polypeptide formation in clusters of β-alanine amino acids by single ion impact
Source: Nat Commun. 2020 Jul 30;11:3818. doi: 10.1038/s41467-020-17653-z (PMC7393107; doi:10.1038/s41467-020-17653-z)
Supplement: Supplementary file 1 — Supplementary Information [file 41467_2020_17653_MOESM1_ESM.pdf]

Supplementary Information: Polypeptide Formation in Clusters of β-Alanine Amino Acids by Single Ion Impact

Rousseau et al.

Supplementary Notes 1. Experiments

The experiments were performed at the low-energy ion beam infrastructure ARIBE of the GANIL facility in Caen, France.[1] A slow beam of He^{2+} ions with an energy of 30 keV was crossed at an angle of 90° with an effusive beam of loosely bound clusters of β-alanine molecules. The He^{2+} ions were produced in an electron cyclotron resonance ion source. The extracted ion beam was mass-selected in a magnetic dipole and an electric pulsing system provided ion beam bunches with a time-width of 0.5 μs at a repetition rate of 5 kHz. After further collimation the beam is transported to the interaction zone, where it interacts with a molecular effusive beam of neutral clusters. The neutral β-alanine clusters were produced in a liquid nitrogen-cooled cluster aggregation source,[2] where β-alanine powder was heated in an oven device to a temperature of $\sim$ 420K. The molecular vapor enters a cooled region containing the buffer gas He, thus inducing cooling, aggregation and production of clusters with an internal temperature of $\sim$ 80K. The size distribution of the weakly bound clusters is rather wide and follows a log-normal distribution. After the ion collision the measured distribution changes and shifts to smaller sizes due to the energy transfer and the evaporation of neutral molecules. Cationic systems, produced in collisions with the He^{2+} ions, were extracted after the ion beam pulse has passed the interaction zone, into a modified Wiley-McLaren linear TOF mass spectrometer.[3] Experimental studies showed that the mass resolution of the TOF-system can be optimized for a given TOF-range (and hence a given range of mass-over-charge ratios) by the choice of the time delay between the collision event and the ion extraction pulse.[4–6] Different ion mass ranges require different delay times. At the end of the free-flight region of the spectrometer (TOF tube of 1 m in length) these products were post-accelerated by a potential of -19 kV and impacted on a conversion plate producing secondary electrons. These are accelerated by the potential difference between the plate and the TOF tube and deviated by a weak magnetic field produced by two Helmholtz coils towards a micro-channel plate detector. This Daly-type detector allowed for an efficient and uniform detection of ions with m/z ratios of up to

$\sim$ 2000, well above the values analyzed in the present study. The m/z values of different product ions are determined by their time-of-flight in the TOF system. These are measured as differences of a start pulse, corresponding to the application of the extraction field to the TOF system, and the moments of arrival of the secondary electrons on the detector. These signals from the detector are stored in an acquisition system which works in an event-by-event mode. This means that the system is activated by the start pulse and stays activated for a chosen time, during which all pulses of detected ions are registered. Such an event is characterized by the number k of detected fragments and their associated time-of-flights.[7] After the storage of these data, the measuring cycle restarts with a frequency of 5 kHz. In the case that the counting rate is very low, i.e. the probability for one collision-event to occur within one ion pulse is $\ll 1$, the observation of several (k) charged fragments in one ion pulse means, with high probability, that in one collision k charged fragments are formed. A so-called k-stop event requires that the fragmenting system carries at least k charges. The measured mass spectra can be represented as inclusive spectra, containing all events independent of the number k, and as k-stop spectra, which add up all ion distributions from events which are characterized by a given number k of product ions (stops). A clear analysis of ion correlations requires an ion detection efficiency which is close to 1. In the present experiment this efficiency is $\sim$ 0.8, thus requiring the correction of accidental correlations. The experimental setup has been described in more detail elsewhere.[2]

Supplementary Figure 1 shows the k-stop ($k \geq 2$) mass spectrum of the cationic products of the collision between 30 keV He^{2+} projectiles and cold neutral clusters of β-alanine molecules. We can observe the formation of tetrapeptides.

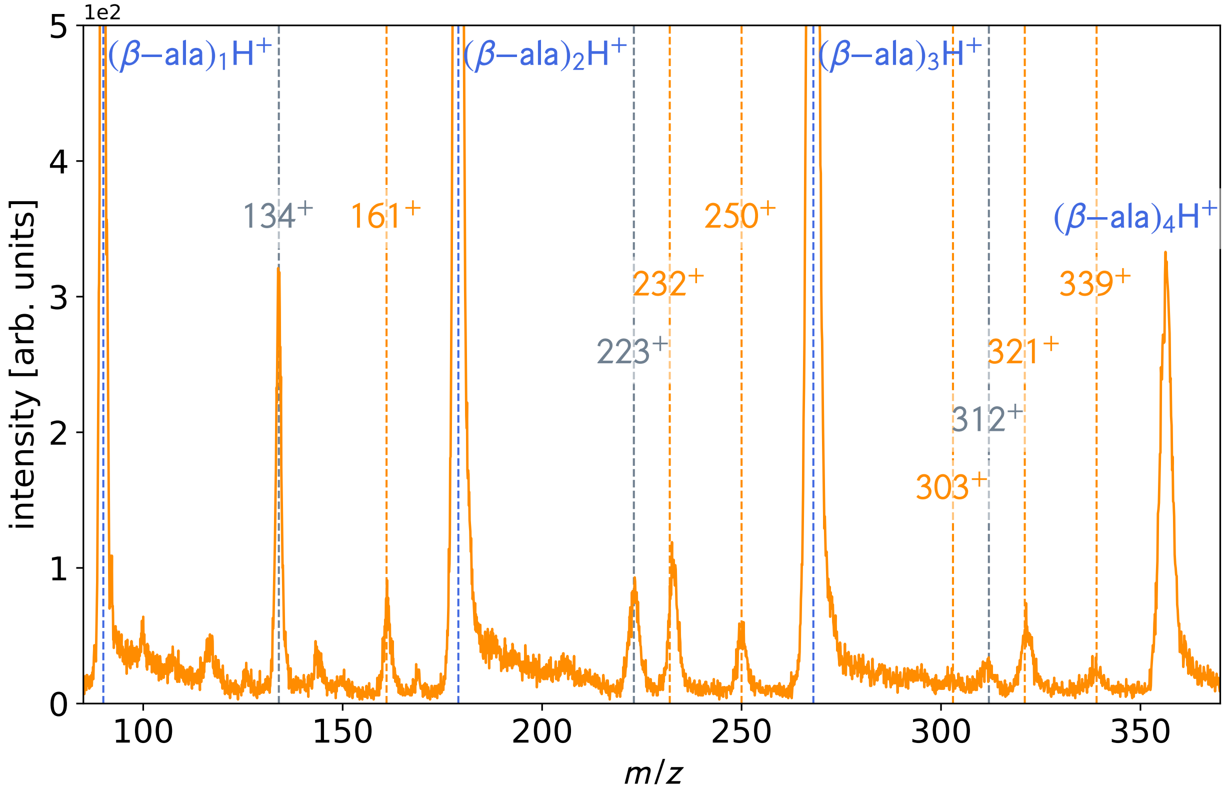

Supplementary Figure 1: Multistop mass spectrum. k-stop ($k \geq 2$) mass spectrum of the cationic products of the collision between 30 keV He^{2+} projectiles and cold neutral clusters of β-alanine molecules. Weakly bound clusters are indicated in blue, while peptide species produced after the collision are in orange and other covalent products are in grey.

Supplementary Notes 2. Theory

Molecular Dynamics (MD) calculations were carried out by using TURBOMOLE.[8] The geometries and energies of the critical points in the Potential Energy Surface (PES) were determined by using the Gaussian09[9] program packages.

Molecular dynamics simulations were performed in the framework of the density functional theory (DFT) in two steps:

1. DFT-based Molecular Dynamics (MD) simulations were carried out using the Born-Oppenheimer Molecular Dynamics (BOMD), in the ground-state electronic potential energy, employing the M06-2X functional[10] in combination with the Ahlrich type basis set SV(P).[11] For long timescale simulations (see Supplementary Table 1) a time-step of $\Delta t = 40$ a.u. (~ 1 fs) and a maximum simulation time of $t_{\max} \sim 9000$ fs were imposed (some trajectories were stopped before, depending on the observed process). The equations of motion were integrated using the Leapfrog Verlet algorithm.[12] The experimental conditions were reflected in the simulations by introducing a certain amount of excitation energy, $E_{\mathrm{exc}} = 2.5 - 12.5$ eV, and extracting one, two or three electrons from the outer electronic shell in a Franck-Condon type transition. The internal energy was randomly distributed over all the nuclear degrees of freedom in each trajectory, (i.e. not projected on the vibrational modes). Simulations were carried out with cluster sizes of 2 to 5 β-alanine molecules and employing starting configurations taken from the most stable neutral conformer.[13] The results of the trajectories performed for the different cluster sizes, ionization degrees, and excitation energies are summarized in Supplementary Table 1. This set of trajectories provides a first approach to identify the most common processes as a function of the cluster size, charge state, and excitation energy.

Supplementary Table 1: Molecular dynamics simulations. Results of the molecular dynamics simulations starting from different excitation energy E_{exc}, charge q, and cluster size n. Propagation has been extended up to the indicated time in each case. Weakly bound molecules (typically through hydrogen bonds) are indicated with $\ldots$, while covalent bonds between molecular fragments are shown with —. In all cases [(β−ala)+H] refers to the β-alanine protonated on the N atom; and [(β−ala)−H] to the deprotonated β-alanine molecule.

	$n = 2$		
	$q = 1$	$q = 2$	$q = 3$
$E_{\mathrm{exc}} = 2.5\,\mathrm{eV}$	$[(\beta\text{−ala})\text{−H}]^0 + [(\beta\text{−ala})\text{+H}]^+$<br>$m/z = 90$<br>$t = 1731\,\mathrm{fs}$	$NH_2CH_2^+ + CH_2CO_2^0 + [(\beta\text{−ala})\text{+H}]^+$<br>$m/z = 30 + 90$<br>$t = 2256\,\mathrm{fs}$	$NH_2CH_2CH_2^+ + COOH^+ + (\beta\text{−ala})^+$<br>$m/z = 44 + 45 + 89$<br>$t = 255\,\mathrm{fs}$
$E_{\mathrm{exc}} = 5.0\,\mathrm{eV}$	$[(\beta\text{−ala})\text{−H} \cdots (\beta\text{−ala})\text{+H}]^+$<br>$m/z = 178$<br>$t = 6142\,\mathrm{fs}$	$NH_2CH_2^+ + CH_2CO_2^0 + [(\beta\text{−ala})\text{+H}]^+$<br>$m/z = 30 + 90$<br>$t = 8653\,\mathrm{fs}$	$(\beta\text{−ala})^{2+} + (\beta\text{−ala})^+$<br>$m/z = 44.5 + 89$<br>$t = 7310\,\mathrm{fs}$
$E_{\mathrm{exc}} = 7.8\,\mathrm{eV}$	$(\beta\text{−ala})^0 + (\beta\text{−ala})^+$<br>$m/z = 89$<br>$t = 1270\,\mathrm{fs}$	$[(\beta\text{−ala})\text{−H}]^+ + [(\beta\text{−ala})\text{+H}]^+$<br>$m/z = 88 + 90$<br>$t = 2602\,\mathrm{fs}$	$NH_2CHCH_3^+ + COOH^+ + (\beta\text{−ala})^+$<br>$m/z = 44 + 45 + 89$<br>$t = 371\,\mathrm{fs}$

	$n = 3$		
	$q = 1$	$q = 2$	$q = 3$
$E_{\mathrm{exc}} = 2.5\,\mathrm{eV}$	$[(\beta{-}\mathrm{ala}){-}\mathrm{H}\cdots(\beta{-}\mathrm{ala}){+}\mathrm{H}\cdots(\beta{-}\mathrm{ala})]^{+}$<br>$m/z = 267$<br>$t = 644\,\mathrm{fs}$	$\mathrm{NH_2CHCH_3^+} + [(\beta{-}\mathrm{ala}){+}\mathrm{H}\cdots(\beta{-}\mathrm{ala})]^{+}$<br>$+\,\mathrm{CO_2^0}$<br>$m/z = 44 + 179$<br>$t = 1043\,\mathrm{fs}$	$(\beta{-}\mathrm{ala})^{+} + (\beta{-}\mathrm{ala})^{+} + (\beta{-}\mathrm{ala})^{+}$<br>$m/z = 89 + 89 + 89$<br>$t = 1122\,\mathrm{fs}$
$E_{\mathrm{exc}} = 5.0\,\mathrm{eV}$	$[(\beta{-}\mathrm{ala}){-}\mathrm{H}\cdots(\beta{-}\mathrm{ala}){+}\mathrm{H}\cdots(\beta{-}\mathrm{ala})]^{+}$<br>$m/z = 267$<br>$t = 978\,\mathrm{fs}$	$[(\beta{-}\mathrm{ala}){-}\mathrm{H}]^{+} + [(\beta{-}\mathrm{ala}){+}\mathrm{H}\cdots(\beta{-}\mathrm{ala})]^{+}$<br>$m/z = 88 + 179$<br>$t = 1412\,\mathrm{fs}$	$(\beta{-}\mathrm{ala})^{+} + (\beta{-}\mathrm{ala})^{+} + (\beta{-}\mathrm{ala})^{+}$<br>$m/z = 89 + 89 + 89$<br>$t = 420\,\mathrm{fs}$
$E_{\mathrm{exc}} = 10.0\,\mathrm{eV}$	$[(\beta{-}\mathrm{ala}){-}\mathrm{H}]^{0} + (\beta{-}\mathrm{ala})^{0} + [(\beta{-}\mathrm{ala}){+}\mathrm{H}]^{+}$<br>$m/z = 89$<br>$t = 3544\,\mathrm{fs}$	$(\beta{-}\mathrm{ala})^{+} + [(\beta{-}\mathrm{ala}){-}\mathrm{H}]^{0} + [(\beta{-}\mathrm{ala}){+}\mathrm{H}]^{+}$<br>$m/z = 89 + 90$<br>$t = 108\,\mathrm{fs}$	$\mathrm{NH_2CH_2^+} + (\beta{-}\mathrm{ala})^{+} + (\beta{-}\mathrm{ala})^{+}$<br>$+\,\mathrm{CH_2COOH^0}$<br>$m/z = 30 + 89 + 89$<br>$t = 3213\,\mathrm{fs}$

	$n = 4$		
	$q = 1$	$q = 2$	$q = 3$
$E_{\mathrm{exc}} = 2.5\,\mathrm{eV}$	$[(\beta\mathrm{-ala})\mathrm{-H}\cdots(\beta\mathrm{-ala})\mathrm{+H}\cdots(\beta\mathrm{-ala})_2]^+$	$\mathrm{NH_2CHCH_3^+} + [(\beta\mathrm{-ala})\mathrm{+H}\cdots(\beta\mathrm{-ala})_2]^+$ $+\,\mathrm{CO_2^0}$	$(\beta\mathrm{-ala})^+ + [(\beta\mathrm{-ala})\mathrm{+H}]^+$ $+\,[(\beta\mathrm{-ala})\mathrm{-H}\cdots(\beta\mathrm{-ala})]^+$
	$m/z = 356$	$m/z = 44 + 268$	$m/z = 89 + 90 + 177$
	$t = 472\,\mathrm{fs}$	$t = 3125\,\mathrm{fs}$	$t = 4263\,\mathrm{fs}$
$E_{\mathrm{exc}} = 5.0\,\mathrm{eV}$	$[(\beta\mathrm{-ala})\mathrm{-H}\cdots(\beta\mathrm{-ala})\mathrm{+H}\cdots(\beta\mathrm{-ala})_2]^+$	$\mathrm{NH_2CHCH_3^+} + [(\beta\mathrm{-ala})\mathrm{+H}\cdots(\beta\mathrm{-ala})_2]^+$ $+\,\mathrm{CO_2^0}$	$\mathrm{NH_2CH_2^+} + (\beta\mathrm{-ala})^+ + [(\beta\mathrm{-ala})\mathrm{+H}]^+$ $+\,\mathrm{CH_2CO_2^0} + (\beta\mathrm{-ala})^0$
	$m/z = 356$	$m/z = 44 + 268$	$m/z = 30 + 89 + 90$
	$t = 3692\,\mathrm{fs}$	$t = 6050\,\mathrm{fs}$	$t = 4163\,\mathrm{fs}$
$E_{\mathrm{exc}} = 10.0\,\mathrm{eV}$	$[(\beta\mathrm{-ala})\mathrm{-H}\cdots(\beta\mathrm{-ala})\mathrm{+H}\cdots(\beta\mathrm{-ala})_2]^+$	$[(\beta\mathrm{-ala})\mathrm{+H}]^+ + [(\beta\mathrm{-ala})\mathrm{+H}\cdots\mathrm{NHCH_2}]^+$ $+\,[(\beta\mathrm{-ala})\mathrm{-H}]^0 + \mathrm{CH_2COOH^0}$	$(\beta\mathrm{-ala})^+ + (\beta\mathrm{-ala})^+$ $+\,[(\beta\mathrm{-ala})\mathrm{-H}\cdots(\beta\mathrm{-ala})\mathrm{+H}]^+$
	$m/z = 89$	$m/z = 89 + 119$	$m/z = 89 + 89 + 178$
	$t = 1270\,\mathrm{fs}$	$t = 2602\,\mathrm{fs}$	$t = 371\,\mathrm{fs}$

	$n=5$		
	$q=1$	$q=2$	$q=3$
$E_{\mathrm{exc}}=2.5\,\mathrm{eV}$	$[(\beta\text{−ala})\text{−H}\cdots(\beta\text{−ala})\text{+H}\cdots(\beta\text{−ala})_3]^+$	$[(\beta\text{−ala})\text{+H}\cdots(\beta\text{−ala})\cdots\mathrm{NHCH_2}]^+$ $+\,[(\beta\text{−ala})\cdots(\beta\text{−ala})\text{+H}]^+ + \mathrm{CH_2CO_2^0}$	$[(\beta\text{−ala})\text{+H}]^+ + [(\beta\text{−ala})\text{+H}]^+$ $+\,[(\beta\text{−ala})\text{+H—NH_2CH_2CH_2}]^+$
	$m/z=445$	$m/z=179+20$	$m/z=90+90+133$
	$t=739\,\mathrm{fs}$	$t=2268\,\mathrm{fs}$	$t=1134\,\mathrm{fs}$
$E_{\mathrm{exc}}=5.0\,\mathrm{eV}$	$[(\beta\text{−ala})\text{−H}\cdots(\beta\text{−ala})\text{+H}\cdots(\beta\text{−ala})_3]^+$	$[(\beta\text{−ala})\text{+H}]^+ + [(\beta\text{−ala})\text{+H}\cdots(\beta\text{−ala})]^+$	$[(\beta\text{−ala})\text{+H}]^+ + [(\beta\text{−ala})\text{+H}]^+$ $+\,[(\beta\text{−ala})\text{−H}\cdots(\beta\text{−ala})\text{+H}]^+$
	$m/z=445$	$m/z=90+179$	$m/z=89+90+178$
	$t=776\,\mathrm{fs}$	$t=2007\,\mathrm{fs}$	$t=886\,\mathrm{fs}$
$E_{\mathrm{exc}}=12.0\,\mathrm{eV}$	$[(\beta\text{−ala})\text{−H}\cdots(\beta\text{−ala})\text{+H}\cdots(\beta\text{−ala})_2]^+$ $+\,(\beta\text{−ala})^0$	$[(\beta\text{−ala})\text{−H}\cdots(\beta\text{−ala})\text{+H}\cdots(\beta\text{−ala})_3]^{2+}$	$[(\beta\text{−ala})\text{+H}]^+ + [(\beta\text{−ala})\text{+H}\cdots(\beta\text{−ala})]^+$ $+\,[(\beta\text{−ala})\text{+H—NH_2CH_2CH_2}]^+$
	$m/z=356$	$m/z=225$	$m/z=90+133+178$
	$t=3473\,\mathrm{fs}$	$t=1151\,\mathrm{fs}$	$t=5254\,\mathrm{fs}$

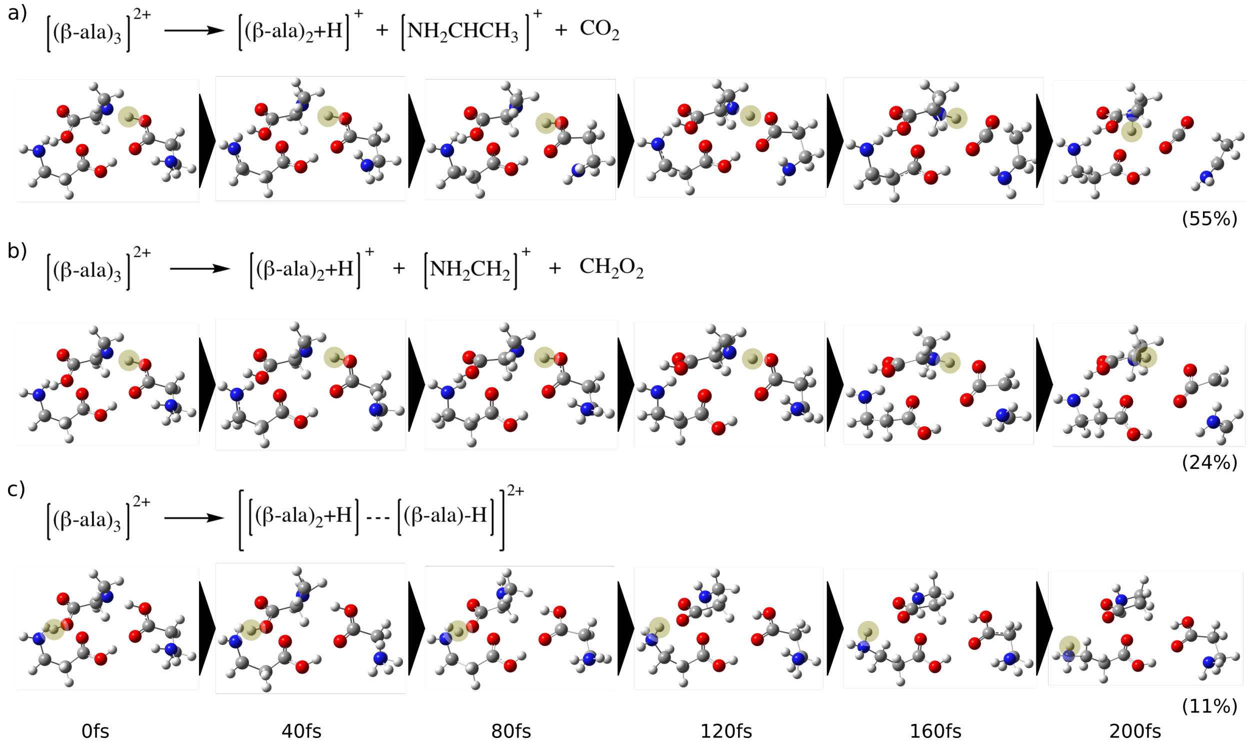

Supplementary Figure 2: Proton transfer in doubly charged trimer. DFT-based molecular dynamics analysis of the doubly charged trimer. The proton transfer is highlighted. H atoms are given in white, C atoms in grey, N atoms in blue, and O atoms in red.

2. The second part of the MD simulations consisted of a statistical analysis considering two different aspects of the observed processes:

   (i) In order to identify the proton transfer, we calculated 100 trajectories starting from the doubly-charged trimer. Results are summarized in Supplementary Figure 2 including examples of trajectories leading to the most probable processes. In all of them, proton transfer leading to the protonated dimer $[(\beta-\text{ala})_2\text{+H}]^+$ is observed. In this figure we not only see very fast intermolecular H migration leading to a protonated dimer (c), but also that this is accompanied by subsequent fragmentation of the deprotonated monomer ($m/z = 88$) into (a)($NH_2CHCH_3^+ + CO_2$), or (b)($NH_2CH_2^+ + CH_2CO_2$).

   (ii) To study the peptide bond formation, we run a set of 100 additional trajectories starting from the singly-charged protonated dimer in the geometry of the key transition state found for the corresponding mechanism. Results of these trajectory calculations

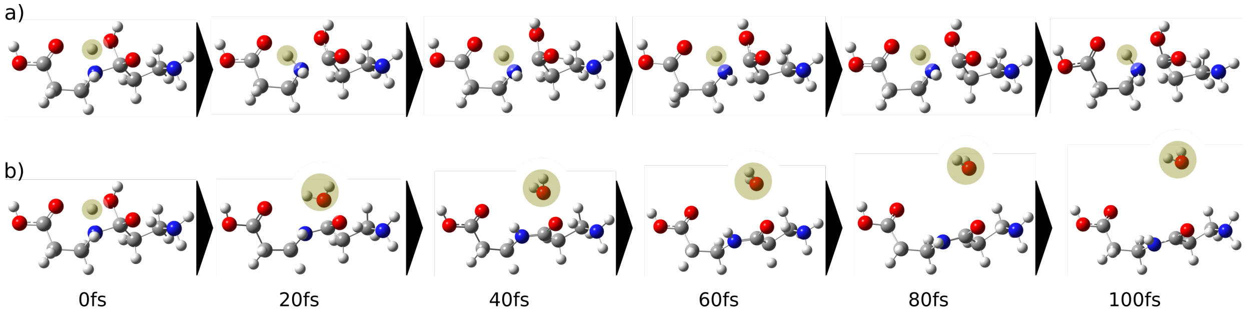

Supplementary Figure 3: Formation of peptide bond in protonated dimer. DFT-based molecular dynamics analysis of the singly charged protonated dimer. The release of the water molecule is highlighted. H atoms are given in white, C atoms in grey, N atoms in blue, and O atoms in red.

are given in Supplementary Figure 3. Half of the trajectories led to peptide bond formation accompanied by water release.

Both MD simulations in (i) and (ii) were performed using the M062X/def2-SVP level of theory with a smaller time-step of $\Delta t = 4$ a.u. (~ 0.1 fs) and 5 eV of internal excitation energy, E_{exc}.

Potential Energy Surface Exploration

Simulations were also carried out for an exhaustive exploration of relevant parts in several potential energy surfaces for different systems. For this, we used, as starting points, the results obtained from the first set of MD simulations. Geometry optimizations have been carried out at the M06-2X/6-311++G(d,p) level of theory (the semi-empirical AM1 level[14] was exceptionally used for the trimer (Figure 5 of the main text) and for two transition states marked with "*" in Figure 3 in the main text. Harmonic vibrational frequencies have been also evaluated at the same level to characterize minima and transition states in the PES and to compute the Zero Point Energy (ZPE) correction. The absence of "imaginary" frequencies in the calculated vibrational spectra was interpreted as the standard condition to define the point on the PES as a minimum. On the other hand, the existence of just one "imaginary" frequency was used as a criterion of the first order transition state geometry achievement. For the obtained transition states, intrinsic reaction coordinate (IRC) calculations have been

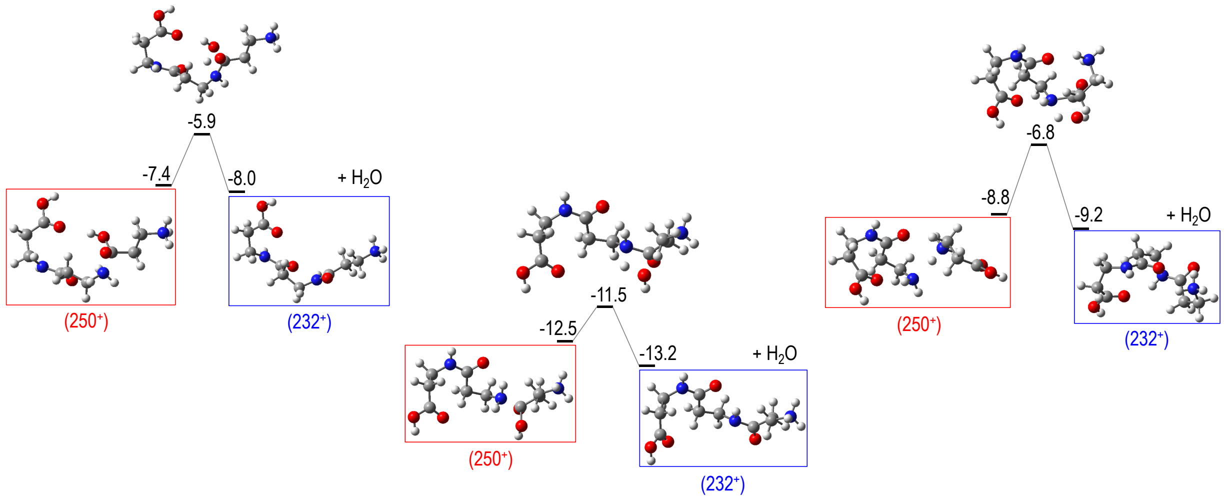

Supplementary Figure 4: Peptide bond formation between a dipeptide and a weakly bound molecule. Tripeptide formation starting from a protonated dipeptide and a loosely bound β-alanine molecule (red boxes). Geometries computed at the AM1 level of theory. Relative energies (in eV) calculated at the DFT-M062X/6-311++G(d,p) level over the previously obtained structure and referred to the most stable neutral trimer are given next the molecular structures. H atoms are given in white, C atoms in grey, N atoms in blue, and O atoms in red.

carried out to verify the minima they connect. More accurate values of the energy were computed with a high ab initio level of theory - Coupled Cluster method including single and double excitations in combination with a triple-z basis set including extra diffuse and polarization functions: CCSD/6-311++G(d,p) level of theory. These calculations were performed over the geometries previously obtained and the final energies were corrected with the Zero Point Energy obtained in the DFT simulations. The relative energies computed at the DFT level are in a very good agreement with those obtained with CCSD.

Additional information on the Potential Energy Surfaces not shown in the main article is given in Supplementary Figures 4 and 5. Tripeptide formation is shown in Supplementary Figure 4. Supplementary Figure 5 shows peptide bond formation starting from the non-protonated β-alanine dimer. The geometry of the structures in these figures was obtained at the AM1 level and final energies with DFT-M062X.

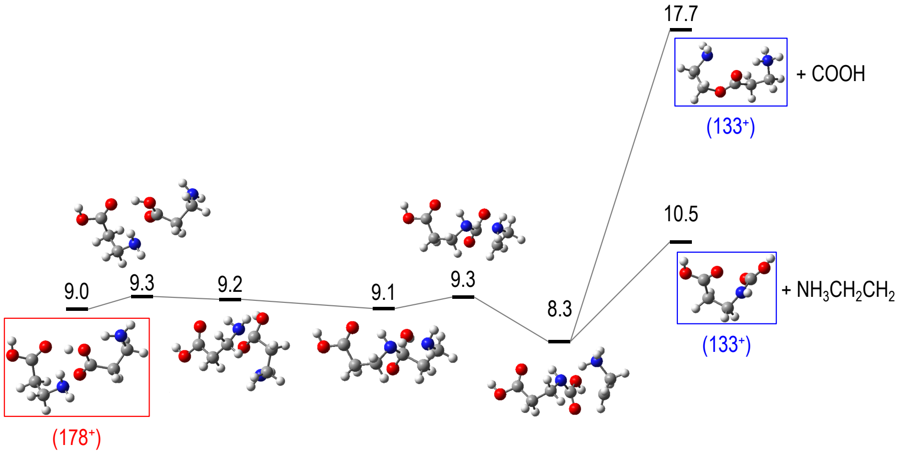

Supplementary Figure 5: Peptide bond formation in a non-protonated dimer. Peptide bond formation starting from the non-protonated β-alanine dimer (red box). Geometries computed at the AM1 level of theory. Relative energies (in eV) calculated at the DFT-M062X/6-311++G(d,p) level over the previously obtained structure and referred to the most stable neutral dimer are given next the molecular structures. H atoms are given in white, C atoms in grey, N atoms in blue, and O atoms in red.

Supplementary Table 2 shows the molecular structure corresponding to the main peaks observed in the experiment.

Supplementary Table 2: Identification of the main covalent products. Characterization of the dominant products in the region between the monomer and the trimer. The structures shown were computed with the DFT simulations (at the DFT-M062X/6-311++G(d,p) level of theory). H atoms are given in white, C atoms in grey, N atoms in blue, and O atoms in red.

m/z	molecular structure(s)	decay pathway(s)
116	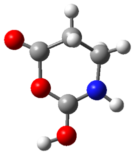	decay of protonated doubly charged dimer (179^{++}) with peptide bond formation by the loss of $NH_3CH_2CH_2^+$ and H_2O
117	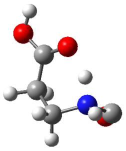	decay of protonated doubly charged dimer (179^{++}) with peptide bond formation by loss of $NH_3CH_2CH_2^+$ and OH

134	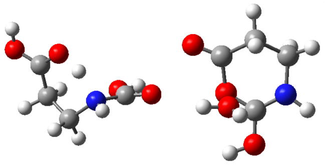	decay of protonated doubly charged dimer (179^{++}) with peptide bond formation by loss of $NH_3CH_2CH_2^+$
144	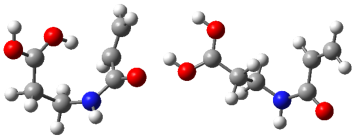	decay of protonated singly charged dimer (179^+) with peptide bond formation by loss of H_2O and NH_3
161	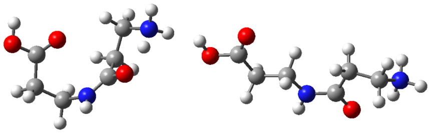	decay of protonated singly charged dimer (179^+) forming a **dipeptide** by loss of H_2O<br>loss of monomer and H_2O from protonated trimer (268^+)
232	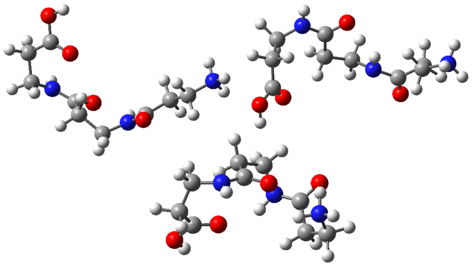	decay of the protonated trimer (268^+), forming in a first step a **dipeptide** by H_2O emission and by the loss of a second H_2O molecule a **tripeptide** with different geometrical structures
250	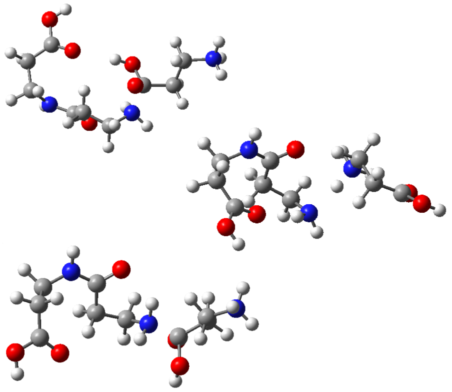	decay of the protonated trimer (268^+) forming a **dipeptide** by the emission of H_2O and loosely bound protonated monomer

References

(1) Bernigaud, V.; Kamalou, O.; Ławicki, A.; Capron, M.; Maisonny, R.; Manil, B.; Maunoury, L.; Rangama, J.; Rousseau, P.; Chesnel, J.-Y. et al. ARIBE: a low-energy ion beam facility in Caen. *Publications of the Astronomical Observatory of Belgrade* **2008**, *84*, 83–86.

(2) Bergen, T.; Biquard, X.; Brenac, A.; Chandezon, F.; Huber, B. A.; Jalabert, D.; Lebius, H.; Maurel, M.; Monnand, E.; Opitz, J. et al. Multiply charged cluster ion crossed-beam apparatus: Multi-ionization of clusters by ion impact. *Review of Scientific Instruments* **1999**, *70*, 3244–3253.

(3) Chandezon, F.; Huber, B. A.; Ristori, C. A new regime Wiley-McLaren time-of-flight mass spectrometer. *Review of Scientific Instruments* **1994**, *65*, 3344–3353.

(4) Wiley, W. C.; McLaren, I. H. Time-of-Flight Mass Spectrometer with Improved Resolution. *Review of Scientific Instruments* **1955**, *26*, 1150–1157.

(5) Colby, S. M.; Reilly, J. P. Space-Velocity Correlation Focusing. *Analytical Chemistry* **1996**, *68*, 1419–1428.

(6) Delaunay, R.; Mika, A.; Domaracka, A.; Huber, B. A.; Rousseau, P. Ion-collision induced molecular growth in polycyclic aromatic hydrocarbon clusters: comparison of $C_{16}H_{10}$ structural isomers. *European Physical Journal D* **2018**, *72*, 149.

(7) Capron, M.; Díaz-Tendero, S.; Maclot, S.; Domaracka, A.; Lattouf, E.; Ławicki, A.; Maisonny, R.; Chesnel, J.-Y.; Méry, A.; Poully, J.-C. et al. A Multicoincidence Study of Fragmentation Dynamics in Collision of γ-Aminobutyric Acid with Low-Energy Ions. *Chemistry - A European Journal* **2012**, *18*, 9321–9332.

(8) TURBOMOLE V6.2 2010, a development of University of Karlsruhe and

Forschungszentrum Karlsruhe GmbH, 1989-2007, TURBOMOLE GmbH, since 2007; available from `http://www.turbomole.com`.

(9) Frisch, M. J.; Trucks, G. W.; Schlegel, H. B.; Scuseria, G. E.; Robb, M. A.; Cheeseman, J. R.; Scalmani, G.; Barone, V.; Mennucci, B.; Petersson, G. A. et al. Gaussian 09 Revision E.01. Gaussian Inc. Wallingford CT 2009.

(10) Zhao, Y.; Truhlar, D. G. The M06 suite of density functionals for main group thermochemistry, thermochemical kinetics, noncovalent interactions, excited states, and transition elements: two new functionals and systematic testing of four M06-class functionals and 12 other functionals. *Theoretical Chemistry Accounts* **2008**, *120*, 215–241.

(11) Schäfer, A.; Horn, H.; Ahlrichs, R. Fully optimized contracted Gaussian basis sets for atoms Li to Kr. *The Journal of Chemical Physics* **1992**, *97*, 2571–2577.

(12) Allen, M. P.; Tildesley, D. J. *Computer Simulation of Liquids*; Oxford University Press, USA, 1987.

(13) Piekarski, D. G.; Díaz-Tendero, S. Structure and stability of clusters of β-alanine in the gas phase: importance of the nature of intermolecular interactions. *Physical Chemistry Chemical Physics* **2017**, *19*, 5465–5476.

(14) Dewar, M. J. S.; Zoebisch, E. G.; Healy, E. F.; Stewart, J. J. P. Development and use of quantum mechanical molecular models. 76. AM1: a new general purpose quantum mechanical molecular model. *Journal of the American Chemical Society* **1985**, *107*, 3902–3909.
